# The health economic impact of cow's milk allergy in childhood: A retrospective cohort study

**DOI:** 10.1002/clt2.12187

**Published:** 2022-08-26

**Authors:** Abbie L. Cawood, R. Meyer, Kate E. Grimshaw, Katy Sorensen, Dionisio Acosta‐Mena, Rebecca J. Stratton

**Affiliations:** ^1^ Institute of Human Nutrition Faculty of Medicine Southampton General Hospital Southampton UK; ^2^ Medical Affairs, Nutricia Ltd, White Horse Business Park Trowbridge UK; ^3^ Department of Paediatrics St Mary's Hospital London UK; ^4^ Dietetic Department Salford Care Organisation Salford Royal NHS Foundation Trust Salford UK; ^5^ Cegedim Health Data, Cegedim Rx London UK

**Keywords:** cow's milk allergy, health economics, infants, infections, paediatrics, primary care

## Abstract

**Background:**

Cow's milk allergy (CMA) is one of the most common food allergies among children. Whilst avoidance of cow's milk protein is the cornerstone of management, further treatment of symptoms including those affecting the gastrointestinal, skin and respiratory systems plus other allergic comorbidities, maybe required. This study aimed to quantify the wider economic impact of CMA and its management in the United Kingdom (UK).

**Methods:**

We conducted a retrospective matched cohort study on children with CMA (diagnosis read code and/or hypoallergenic formula prescription for ≥3 months) examining healthcare data (medication prescriptions and healthcare professional contacts) from case records within The Health Improvement Network (A Cegedim Proprietary Database) in the UK. A comparative cost analysis was calculated based on healthcare tariff and unit costs in the UK.

**Results:**

6998 children (54% male; mean observation period 4.2 years) were included (*n* = 3499 with CMA, mean age at diagnosis 4.04 months; *n* = 3499 matched controls without CMA). Compared to those without CMA, medications were prescribed to significantly more children with CMA (*p* < 0.001) at a higher rate (*p* < 0.001). Children with CMA also required significantly more healthcare contacts (*p* < 0.001) at higher rate (*p* < 0.001) compared to those without CMA. CMA was associated with additional potential healthcare costs of £1381.53 per person per year.

**Conclusion:**

The findings of this large cohort study suggest that CMA and its associated co‐morbidities presents a significant additional healthcare burden with economic impact due to higher prescribing of additional medications. Further research into management approaches that may impact these clinical and economic outcomes of CMA is warranted.

## 1 INTRODUCTION

Cow's milk allergy (CMA) is one of the most common childhood food allergies, affecting 2%–5% of infants in Europe.^1^, ^2^, ^3^, ^4^ It is defined as a reproducible, immune‐mediated response to one or more of the proteins which mainly constitute whey and casein in cow's milk.^1^, ^2^, ^5^ There are different types of CMA, classified by the mechanism and timing of the immune reaction and associated its symptoms. Immunoglobulin E (IgE) mediated CMA is estimated to account for around 44% of CMA cases according to Euro‐Prevall birth cohort data.^3^ It produces specific IgE antibodies upon exposure to cow's milk protein (CMP), which triggers immediate onset of symptoms, typically within minutes to an hour of exposure. These symptoms may affect multiple organ systems, including the skin, gastrointestinal and respiratory systems, with anaphylactic shock occurring in severe cases.^2^ Conversely, non‐IgE mediated CMA, may contribute up to 56% of cases according to the same cohort study^3^ and is associated with a delayed reaction, manifesting hours or days after CMP exposure. Associated symptoms are more difficult to determine due to their delayed onset and overlap with paediatric functional gastrointestinal disorders, but are considered to predominantly affect the skin and GI system.^2^

Management of CMA necessitates the exclusion of CMP from the diet. Whilst breastmilk remains the ideal nutrient source in infants with CMA, infants who are not exclusively breastfed require a hypoallergenic formula (HAF), which includes extensively hydrolysed formulas (eHF), hydrolysed rice formulas (HRF) or amino acid formulas (AAF).^1^, ^2^, ^6^ eHF or HRF, where available, are considered to be appropriate first‐line in the majority of formula‐fed infants with CMA.^1^, ^2^, ^6^, ^7^ However, in severe cases, where symptoms remain unresolved with eHF or HRF, or where anaphylaxis, faltering growth and/or multiple systems are involved, an AAF may be required.^1^, ^2^, ^6^, ^7^ Treatment of presenting symptoms and allergic co‐morbidities may also require the use of a range of medications, such as steroid creams, emollients, antacids, H_2_ antagonists, proton pump inhibitors, and adrenaline autoinjectors.^8^ Medications for the treatment of allergic conditions have been estimated to account for 11% of the primary care prescribing budget in the United Kingdom (UK).^9^ Additional costs have been attributed to the demands of allergic conditions on healthcare services, including general practice consultations, hospital admissions and appointments with dietitians and other specialists.^8^, ^9^ Studies have also reported increased incidence of, and susceptibility to, infections in allergic conditions,^10^, ^11^, ^12^, ^13^, ^14^, ^15^ which might come with further implications for the costs of care.

Whilst previous studies^8^ have estimated the impact of CMA and its associated costs, this retrospective cohort study aims to compare the healthcare usage, including prescriptions, general practice contacts, dietetic contacts, specialist referrals and hospital admissions, of children with CMA to those without, from a contemporaneous national dataset. A comparative cost analysis was also conducted.

## 2 MATERIALS AND METHODS

### 2.1 Study design

We conducted a retrospective cohort study comparing case records extracted from The Health Improvement Network (THIN, A Cegedim Proprietary Database) of children with CMA compared to children without CMA in the UK. Similar retrospective research methods using the THIN database have been used in over 1000 published studies.^16^

### 2.2 The Health Improvement Network (THIN)

At the time of data extraction, 2.9 million anonymised active patient records from approximately 365 general practices were contained within the THIN database, which has been found to be generalisable to the UK population.^17^ Within these records, patient demographics and clinical history, including symptoms, diagnoses, procedures, healthcare professional referrals and consultations, are recorded as read‐codes. Read‐codes have been in use as a coded thesaurus of clinical terms for healthcare professionals since 1985.^18^ Medication prescription data is recorded within case records using the World Health Organisation index of Anatomical Therapeutic Chemical (ATC) codes.^19^

### 2.3 Study population

Data was extracted on the 4^th^ November 2020 from 6998 anonymised case records indexed within the last 5 years (data from 2015 to 2020). This included 3499 children with confirmed or suspected CMA at ≤ 12 months of age. Confirmed CMA was defined by a CMA diagnosis read‐code. In the absence of this specific read‐code, suspected CMA was defined by the prescription of a HAF for at least three consecutive months, in order to exclude children who had received a short‐term HAF prescription for CMA diagnostic purposes.^20^ A cohort of 3499 children without CMA (matched for age, sex and Index of Multiple Deprivation [IMD: quintiles 1 = least deprived to 5 = most deprived, calculated from the IMD score distribution])^21^, ^22^, ^23^, ^24^ were also included. Exclusion criteria aimed to exclude children receiving HAF for documented conditions other than allergy, and those with conditions which could confound clinical outcomes, including:

- Children with read‐codes for intestinal failure; necrotizing enterocolitis; cancer, malignancy or tumour; congenital heart disease; cystic fibrosis; cerebral palsy; metabolic conditions; chromosomal anomalies
- Children prescribed any other medical nutrition product not indicated for CMA

### 2.4 Ethical approval

Ethical approval for this study was granted by the Scientific Review Committee which approves research using the THIN database (protocol reference number: 20‐009).

### 2.5 Study variables and outcome measures

Data were extracted from case records using pre‐defined read‐codes and ATC codes. Demographic data included age, sex, location, IMD, ethnicity, presence of other allergies and family history of allergies. Data on healthcare resource usage included GP contacts, Dietitian contacts, specialist referrals, hospital admissions (including emergency department admissions), prescriptions for HAF, antibiotics, dermatologicals, anti‐reflux medications, inhalers and adrenaline.

### 2.6 Statistical analysis

Healthcare outcomes were measured from birth over the duration of available data for each child (referred to as the observation period throughout). Results were presented as the number and percentage of children who had the outcome at least once during their observation period, and as the outcome rate per 5‐person‐years. This provided an estimate of the average number of times that a child in the cohort would be affected by the outcome within 5 years. Rates per 5‐person‐years were calculated by dividing the total number of instances of a specific outcome by the total number of years over which the children were observed during the study, then multiplying by five.

Between‐group differences for proportional data were measured using Fisher's exact or chi‐square test of independence, where appropriate. The Poisson test was used to measure between‐group differences in rates. Statistical significance was set at *p* < 0.05. All statistical analysis was performed using *R* software version 4.0.2.^25^

### 2.7 Cost analysis

A comparative cost analysis was used to compare the healthcare costs of children with CMA to those of children without CMA. Individual costs included prescriptions (HAF, dermatological medications, anti‐reflux medications, inhalers, adrenaline, and antibiotics) and healthcare contacts (GP, Dietitian, other paediatric allergy specialists, and all cause hospital admissions) (Table 1).

**TABLE 1:** National Healthcare Service cost estimates used in the comparative cost analysis^26^, ^27^, ^28^, ^29^

	Cost per infant per item
Prescriptions	
HAF (per 400 g tin)^a^	£14.46
Dermatological (soft paraffin)^a^	£3.76
Anti‐reflux (ranitidine^b^)^c^	£0.83
Inhalers (salbutamol)^c^	£1.85
Adrenaline auto‐injectors (Epipen Jr)^c^	£59.88
Antibiotics (amoxicillin)^c^	£0.83
Healthcare contacts	
GP^d^	£39
Dietitian^e^	£92
Other specialist (consultant paediatrician)^f^	£237
Hospital admission^g^	£577.33

Abbreviations: HAF, hypoallergenic formulae; GP, general practitioner.

^a^ Calculated weighted mean cost of all 400 g tins of eHF and AAF powders available on prescription in the UK at time of data extraction.^26^

^b^ Which was still available during the observation period.

^c^ Based on the most commonly used, assuming the lowest cost from range, “Individual Preparations” Section.^27^

^d^ Based on unit cost per surgery consultation lasting 9.22 min.^28^

^e^ Based on unit cost for a dietitian appointment (group session or one‐to‐one).^28^

^f^ Based on unit cost for average paediatric consultant‐led outpatient attendance, assuming referral led to one appointment.^28^

^g^ Calculated average non‐elective spell tariff for paediatric hospital admission for upper respiratory tract infections.^29^

The costs for prescribing HAF were calculated as a weighted mean of the prices listed on the Monthly Index of Medical Specialties^26^ for all 400 g tins of eHF and AAF powders available on prescription in the UK at the time of data extraction. This was based on the estimated mean HAF intake among the CMA cohort, calculated from prescription dosage and duration data, and accounting for proportionate usage of eHF and AAF among the cohort. The costs for medication prescriptions were obtained from the England Prescription Cost Analysis,^27^ a very conservative cost was used, using the national ingredient costs per item (NIC), based on the lowest costing medication of the most commonly used medications, for each type of medication, for the whole cohort, during the observation period. For dermatologicals this was paraffin; for anti‐reflux, ranitidine; for inhalers, salbutamol; for adrenaline, Epipen Jr; and for antibiotics it was amoxicillin.

Individual costs for GP, dietitian and other paediatric allergy specialist contacts were obtained from Unit Costs of Health and Social Care 2020.^28^ The latter was based on referral data within the THIN database, conservatively based on the type of paediatric allergy specialist with the lowest unit cost, and assumed that each referral led to one appointment and no follow up. Individual costs for hospital admissions were obtained from the 2020/2021 National Tariff Payment System.^29^ In the absence of data relating to reason for hospital admission, these costs were presented as a mean of the range of paediatric admissions costs for the most common type of infection (respiratory) documented among the cohort, reported elsewhere.^30^

Unit costs for each healthcare resource were extrapolated to the respective healthcare usage rates, presented per person‐year and per 5‐person‐years, to give an indication of CMA‐associated healthcare costs over 1 year, and 5 years, of early life.

## 3 RESULTS

### 3.1 Characteristics

The entire cohort was observed for a mean period of 4.2 years (range 3.5–5.8 years). Groups were well matched for age, sex and level of deprivation (Table 2). There were statistically significant differences between groups in location and ethnicity.

**TABLE 2:** Baseline characteristics

Characteristic	CMA	non‐CMA	*p*‐value
Male, *n* (%)	1896 (54%)	1896 (54%)	>0.9
Location, *n* (%)			
England	968 (28%)	1285 (37%)	<0.001
Northern Ireland	607 (17%)	385 (11%)	
Scotland	978 (28%)	1033 (30%)	
Wales	946 (27%)	796 (23%)	
IMD quintile, *n* (%)			
5th	776 (23%)	788 (23%)	0.071
4th	916 (27%)	915 (27%)	
3rd	597 (18%)	546 (16%)	
2nd	378 (11%)	449 (13%)	
1st	743 (22%)	726 (21%)	
Ethnicity, *n* (%)			
White	1207 (93%)	1265 (87%)	<0.001
Mixed/multiple ethnic groups	17 (1.3%)	31 (2.1%)	
Asian/Asian British	51 (3.9%)	85 (5.8%)	
Black/Black British	19 (1.5%)	59 (4.0%)	
Other	10 (0.8%)	17 (1.2%)	
Presence of ‘other’ allergy	547 (16%)	184 (5.3%)	<0.001
Family history of allergy, *n* (%)	55 (1.6%)	25 (0.7%)	0.001

Abbreviations: 1st, least deprived; 5th, most deprived; CMA, cow’s milk allergy; IMD, index of multiple deprivation.

Of the CMA group, 29% had a CMA read‐code (all of whom were prescribed HAF), with the remainder assigned to this group due to having at least three consecutive months of HAF prescription. The mean age of CMA diagnosis (defined as age at entry of a CMA read‐code or first hypoallergenic formula prescription) was 4.04 (SD 2.79) months. Of the CMA cohort, 100% were prescribed HAF (mean 122 [±35.6] g/day), for a mean of 9.5 (±9.1) months; of whom 88% were prescribed eHF, and 35% AAF, indicating that some children had been prescribed both types of formula during the observation period.

### 3.2 Healthcare usage

Healthcare usage (including medication prescriptions, healthcare professional contacts and hospital admissions) was significantly higher among the CMA cohort than the non‐CMA cohort (Table 3). Only a small proportion of children did not have prescriptions for antibiotics, anti‐reflux medications, dermatologicals, inhalers and adrenaline, 1.2% of the CMA cohort and 9% of the non‐CMA cohort (*p* < 0.001). Additionally, significantly more children with CMA had contacts with the GP, referrals to the dietitian and other specialists, and hospital admissions. Per 5‐person‐years, the rates of all healthcare usage were significantly higher among children with CMA compared to those without.

**TABLE 3:** Differences in healthcare usage among children with CMA versus those without CMA

	CMA	Non‐CMA	*p*‐value
Medication prescriptions			
Antibiotics			
*n* (%) of children^a^	3036 (87%)	2684 (77%)	<0.001
Prescription rate^b^	6.750	4.490	<0.001
Anti‐reflux medications			
*n* (%) of children^a^	2164 (62%)	564 (16%)	<0.001
Prescription rate^b^	5.540	0.925	<0.001
Dermatological medications			
*n* (%) of children^a^	3002 (86%)	2460 (70%)	<0.001
Prescription rate^b^	10.105	5.185	<0.001
Inhalers			
*n* (%) of children^a^	1448 (41%)	1030 (29%)	<0.001
Prescription rate^b^	2.615	1.450	<0.001
Adrenaline			
*n* (%) of children^a^	122 (3.5%)	19 (0.5%)	<0.001
Prescription rate^b^	0.195	0.030	<0.001
*None of the above prescriptions, n* (%)	41 (1.2%)	314 (9.0%)	<0.001
Healthcare contacts			
GP contacts (clinic/home visit/phone)			
*n* (%) of children^a^	154 (4.4%)	0.150	<0.001
GP contact rate^b^	99 (2.8%)	0.100	<0.001
Dietitian contacts			
*n* (%) of children^a^	689 (20%)	50 (1.4%)	<0.001
Dietitian contact rate^b^	0.475	0.030	<0.001
Other specialist referrals			
*n* (%) of children^a^	260 (7.4%)	107 (3.1%)	<0.001
Specialist referral rate^b^	0.120	0.045	<0.001
Hospital admissions			
*n* (%) of children^a^	2012 (58%)	1609 (46%)	<0.001
Hospital admission rate^b^	2.220	1.460	<0.001

Abbreviations: CMA, cow's milk allergy; GP, general practice.

^a^ Percentage of children with at least one occurrence during observation period.

^b^ per 5‐person‐years.

### 3.3 Comparative cost analysis

A comparative analysis of healthcare unit costs (Table 1) and usage rates (Table 3) for each cohort found that CMA was associated with additional healthcare costs. Children with CMA were estimated to generate £1559.27 per person‐year in CMA‐associated healthcare costs, equating to £7796.34 over 5‐person‐years. Children without CMA were estimated to generate £177.74 per person‐year, reaching £888.70 over 5‐person‐years. This equates to a difference in healthcare costs of £1381.53 per person‐year, and £6907.64 per 5‐person‐years.

When assuming a 2.5% prevalence from the estimated 2%–5% CMA prevalence range described elsewhere,^1^, ^2^, ^3^, ^4^ extrapolation to the UK infant population^31^ suggests that CMA may account for additional healthcare costs of more than £25.7 million per year, which could exceed £128.7 million over 5 years, across the UK.

## 4 DISCUSSION

To our knowledge, this is the largest UK cohort study to compare the healthcare and economic impact of children with CMA to children without. Nearly 7000 case records contributed 3.5–5.8 years of data to this retrospective analysis, providing valuable insights into the burden of CMA management in the UK.

This study found that children with CMA used significantly more healthcare resources, including medication prescriptions and healthcare contacts, than those without. In particular, prescriptions of anti‐reflux medication increased by nearly 500%. As clinical guidelines do not recommend the use of either H2 antagonists or proton pump inhibitors in children with gastroesophageal reflux disease first‐line, this may support the findings of other studies indicating an over‐prescription of anti‐reflux medication in this population.^32^, ^33^ Prescriptions for dermatological medications and inhalers increased by 95% and 80% respectively. Given the multitude of GI, skin and respiratory symptoms which are common in CMA, it is not surprising that the prescription rates of these medications are increased. This supports the notion of a greater clinical burden of CMA, including a recent publication, of significantly increased GI, skin and respiratory symptom rates observed among children with CMA compared to those without.^30^

A novel and important finding of this study related to the prescription of antibiotic medications, which were prescribed to significantly more children with CMA, and at a 50% higher rate, compared to children without, which is suggestive of a greater infectious burden. Indeed, the link between allergic and infectious illness has been discussed previously.^34^, ^35^ Studies have shown an increased susceptibility to infections among children^36^ and adults^37^ with allergy, compared to those without. Children with allergic conditions have been found to have an increased incidence of ear infections^10^, ^11^, ^13^, ^38^ and frequent upper respiratory tract infections.^14^ Recently, significantly higher rates of GI, skin, ear and respiratory infections have been documented among children with CMA, compared to those without, increasing by 62%, 37%, 44% and 37% respectively.^30^

A number of causal mechanisms may be involved in the link between allergy and infection. Allergic inflammation has been hypothesised to impair the action of antiviral cytokines, leading to a delayed immune response and the recurrence of infections in allergic rhinitis^39^ and atopic asthma.^34^ Irregular levels of immune cells and antibodies such as lymphocytes, immunoglobulin‐A and immunoglobulin‐G subclasses are also thought to contribute to an increased susceptibility to infection among children with food protein induced gastrointestinal allergies and multiple food allergies.^14^, ^15^ Not least, the development and maintenance of the immune system may be mediated by the commensal gut bacteria,^40^, ^41^ which has been found to be dysbiotic among infants with CMA.^42^, ^43^, ^44^, ^45^, ^46^ Modification of the gut microbiome has been associated with a reduction of infections and antibiotic prescriptions,^45^ and invites consideration of management strategies to address this potential therapeutic target in allergic children.

In addition to the significant increases in medication prescriptions, the present study also found significantly increased rates of healthcare contacts among children with CMA, equating to 50% more GP contacts, 167% more specialist referrals and 52% more hospital admissions, than those without. Most markedly, dietetic contacts increased by more than 1400% among children with CMA compared to those without. Current UK guidelines recommend the involvement of a dietitian in CMA management,^1^ and allergy accounts for a substantial proportion of a paediatric dietetic caseload in clinical practice, which may offer some explanation as to the scale of this difference between groups.

Overall, the increases in healthcare usage observed among infants with CMA was associated with an annual healthcare cost more than eight times greater than that of an infant without CMA, which exceeded £25.7 million per year across the UK. This is consistent with previous research demonstrating the extensive impact of allergic conditions on UK healthcare services and associated costs.^8^, ^9^ One study which modelled costs based on data from 1000 infants during their first 12 months from initial presentation estimated a £25.6 million cost to the National Health Service (NHS),^8^ based on 2006/07 unit resource costs. There are some methodological differences between studies which may account for these similar cost estimates despite the time difference of the studies (described below). However, if we compared like for like the cost of just the CMA cohort (£1559.27pp) as opposed to the difference between the two cohorts (£1381.53pp), the costs would extrapolate to £29.1 million per year across the UK. This study has some potential limitations. A conservative approach to cost‐analysis using the lowest medication NIC and healthcare unit costs was used, which did not address potential differences among subclass medication groups which may warrant further investigation. Also the methods of measuring healthcare usage did not account for other diagnostic tests, consultations or medication prescriptions that may have occurred within the cohorts, and may not have been adequately sensitive to detect specialist care which took place outside of primary care, as interactions between primary and secondary care are captured in different ways. Although we quantified all possible specialist events, read codes focussed specifically on the dietitian. This along with other key methodological differences, including the type of study (computer generated model following presentation vs. matched cohort study once on HAF), inclusion criteria (read code for CMA and at least 1 prescription of HAF vs. read code for CMA ±3 months or HAF prescription), reference costs used, and presenting costs differently (total costs vs. extra costs), may all potentially explain why the cost of CMA in the present study was similar that reported more than a decade ago^8^ Sub‐group analysis to assess whether differences in health care use and costs (either directly or indirectly related) existed for children with confirmed (29% of group) or suspected (71% of group) CMA was not performed as part of this study but future exploration of this kind is needed to provide further valuable insights.

One of the other potential limitations of this study is that there was a significant difference in terms of location (country of residence) and ethnicity between the two cohorts as the groups were not matched for these variables. Further exploration is needed to ascertain any potential reasons for such differences between CMA and non‐CMA cohorts and to investigate in future analysis the potential impact of location and ethnicity in the UK on the results on health care use and costs.

Thirdly, variations in recording practices may have led to differences in the data shown in the present study, and that reported elsewhere. For example, family history of allergy and other allergies are recognised as risk factors for CMA,^20^ but were reported in only a small number of cases in the CMA cohort. The rates of GP contacts in the present study were lower than might be expected, as allergic conditions may account for over 12.5 million GP consultations per year.^9^ In the present study, GP contacts may have been documented as read‐codes relating to the reason for, or outcome of, the contact, such as the diagnosis or medication prescription. This may have led to an underestimation of the general practice burden of CMA, and associated costs. However, a similar margin of error is likely to apply to both groups, affecting data recorded but not necessarily the relative differences between groups.

These variations in recording practices also necessitated a pragmatic approach to recruitment. In the absence of CMA diagnostic read‐codes, children were included in the CMA cohort if they had a HAF prescription for at least three consecutive months. Whilst the full eligibility criteria was likely to exclude children receiving HAF for conditions other than allergy,^47^ or as an elimination diet for diagnostic purposes,^20^ this resulted in 100% of the CMA cohort having current or historic HAF prescriptions. This may exceed the rates of HAF prescription typically observed in clinical practice, as breastmilk is recommended as the optimal nutrient source for infants with CMA.^1^, ^2^, ^6^ The cost of HAF as a proportion of the total cost of CMA may therefore have been overestimated. HAF prescription has previously been estimated to account for 38% of the costs of CMA,^8^ although this was higher in the present study. More advanced health‐economic modelling is warranted, along with consideration of strategies which aim to shorten the clinical course of symptoms, and the duration for which HAF is required, in the clinical management of CMA.

## 5 CONCLUSIONS

This large cohort study provides novel evidence of a significant health economic burden of CMA in children. In order to support the advancement of management strategies for children with CMA, further research is required to investigate the clinical phenotypes and management approaches that may impact clinical and health economic outcomes.

## AUTHOR CONTRIBUTIONS

Conceptualization, K. Sorensen, A. L. Cawood, and R. J. Stratton; methodology, K. Sorensen, A. L. Cawood and D. Acosta‐Mena; formal analysis, D. Acosta‐Mena; data curation, K. Sorensen, A. L. Cawood and D. Acosta‐Mena; writing—original draft preparation, K. Sorensen, A. L. Cawood; writing—review and editing, K. Sorensen, A. L. Cawood, R. Meyer, K. E. Grimshaw, D. Acosta‐Mena and R. J. Stratton. All authors have read and agreed to the published version of the manuscript.

## CONFLICTS OF INTEREST

R. Meyer and K. E. Grimshaw have previously received honoraria from Nutricia, Nestle Health Science, Mead Johnson and Abbott. D. Acosta‐Mena is an honorary Associate Professor at the Institute of Health Informatics, University College London, UK, and an employee of Cegedim Rx, who was funded by Nutricia Ltd. to undertake the research. K. Sorensen was previously employed by Nutricia Ltd. A. L. Cawood and R. J. Stratton, both of whom hold honorary research posts with the University of Southampton, are also employed part‐time by Nutricia Ltd.
